# Oral Manifestations of Pediatric Multisystem Inflammatory Syndrome

**DOI:** 10.1111/scd.70082

**Published:** 2025-09-14

**Authors:** Angela Maira Guimarães, Waleska Tychanowicz Kolodziejwski, Camila Adriane Leffa, Priscila Queiroz Mattos da Silva, Juliana Lucena Schussel, Melissa Rodrigues de Araujo

**Affiliations:** ^1^ Department of Stomatology Federal University of Paraná Curitiba Paraná Brazil

**Keywords:** COVID‐19, low‐level light therapy, oral manifestations

## Abstract

**Background:**

Pediatric multisystem inflammatory syndrome (MIS‐C) is a rare condition associated with COVID‐19, with few reports on its oral manifestations.

**Case Report:**

A 13‐year‐old male pediatric patient with presented with fever, diarrhea, odynophagia, conjunctival hyperemia, rash, and bipalpebral edema. On physical examination, he exhibited diffuse ulcerated lesions with crusts on the vermilion border and labial mucosa, primarily affecting the labial commissures. Initially, immune‐mediated diseases such as erythema multiforme or Stevens–Johnson syndrome were suspected, and treatment with topical corticosteroids and photobiomodulation was initiated. However, the lesions worsened, and polymerase chain reaction (PCR) testing of a sample from the lesion confirmed herpes simplex virus type 1. Given the diagnosis, corticosteroid therapy was discontinued, and decontamination using antimicrobial photodynamic therapy (methylene blue 0.01%, 5 min, 660 nm, 100 mW, 6 J/point, energy density of 199.98 J/cm^2^) was performed. However, oral lesions and overall systemic condition worsened, and the patient died.

**Conclusion:**

This case highlights the need for a differential diagnosis of oral lesions in MIS‐C in order to carry out the most appropriate treatment.

## 1 Introduction

During the early months of the COVID‐19 pandemic, it was observed that children generally exhibited milder or asymptomatic forms of the disease, resulting in low hospitalization and mortality rates in this age group [1, 2]. However, this characteristic changed over time. Since 2020, several countries in Europe and North America have reported cases of severe Pediatric multisystem inflammatory syndrome (MIS‐C) associated with COVID‐19 in young patients [1, 3].

According to the Centers for Disease Control and Prevention (CDC), MIS‐C is diagnosed in patients under 21 years of age who present with persistent fever for at least 24 h, signs of inflammation detected in laboratory tests, and involvement of at least two organ systems. Additionally, the patient must have tested positive for SARS‐CoV‐2 or had recent contact with an infected person [3]. Studies have identified other common findings in MIS‐C, with high fever (≥ 38.0°C) for at least five days being one of the most frequent symptoms [1, 2, 3]. Gastrointestinal manifestations such as abdominal pain, vomiting, and diarrhea are also common, as well as cardiovascular alterations, including tachycardia, hypotension, and myocarditis [1].

The oral manifestations of MIS‐C are characterized by erythematous, dry, and/or cracked lips, often causing pain and a burning sensation, in addition to erythema on the dorsal surface of the tongue, known as “strawberry tongue” [1, 2, 3]. Ulcers similar to aphthous stomatitis may also occur, although less frequently [3]. Halepas et al. suggest that oral manifestations may be among the first signs of MIS‐C, highlighting the importance of trained dental professionals in recognizing and diagnosing these lesions in hospitals and healthcare settings [2, 3].

The objective of this case report is to present the oral manifestations and the dental approaches adopted in a child diagnosed with MIS‐C.

## 2 Case Report

A 13‐year‐old male patient, with a history of obesity (BMI ≈ 27) and anxiety, was referred to the hospital for clinical evaluation with a diagnostic hypothesis of Kawasaki disease or macrophage activation syndrome. The patient lived in a rural area and was primarily cared for by his grandparents. Upon admission to the pediatric intensive care unit (PICU), he presented with pancytopenia (hemoglobin: 7.8 g/dL; leukocytes: 0.463 × 10^3^/µL; platelets: 2.900/µL), fever, diarrhea, odynophagia, conjunctival hyperemia, rash, and bipalpebral edema, along with oral lesions. His physical examination revealed tachypnea, hypochromia (+2), dehydration (+), obesity, and a blood pressure of 129/58 mmHg (81 mmHg MAP). Despite these symptoms, on the day of admission (D0), the patient was alert, oriented, and able to communicate normally. Laboratory tests were positive for SARS‐CoV‐2 IgG antibodies.

Initial pharmacological management was promptly initiated following PICU admission. The patient received antifungal prophylaxis with fluconazole (3 mg/kg/day) starting on day 0 and continued throughout the hospitalization. Broad‐spectrum empirical antimicrobial coverage was instituted, including vancomycin, cefepime, metronidazole, and trimethoprim‐sulfamethoxazole, following institutional sepsis protocols. Supportive measures included enteral nutritional support and high‐dose intravenous methylprednisolone pulse therapy (1 g/day for three consecutive days), alongside albendazole and aspirin (80 mg/kg/day). Intravenous immunoglobulin (IVIG) at 2 g/kg was also administered.

**FIGURE 1:**
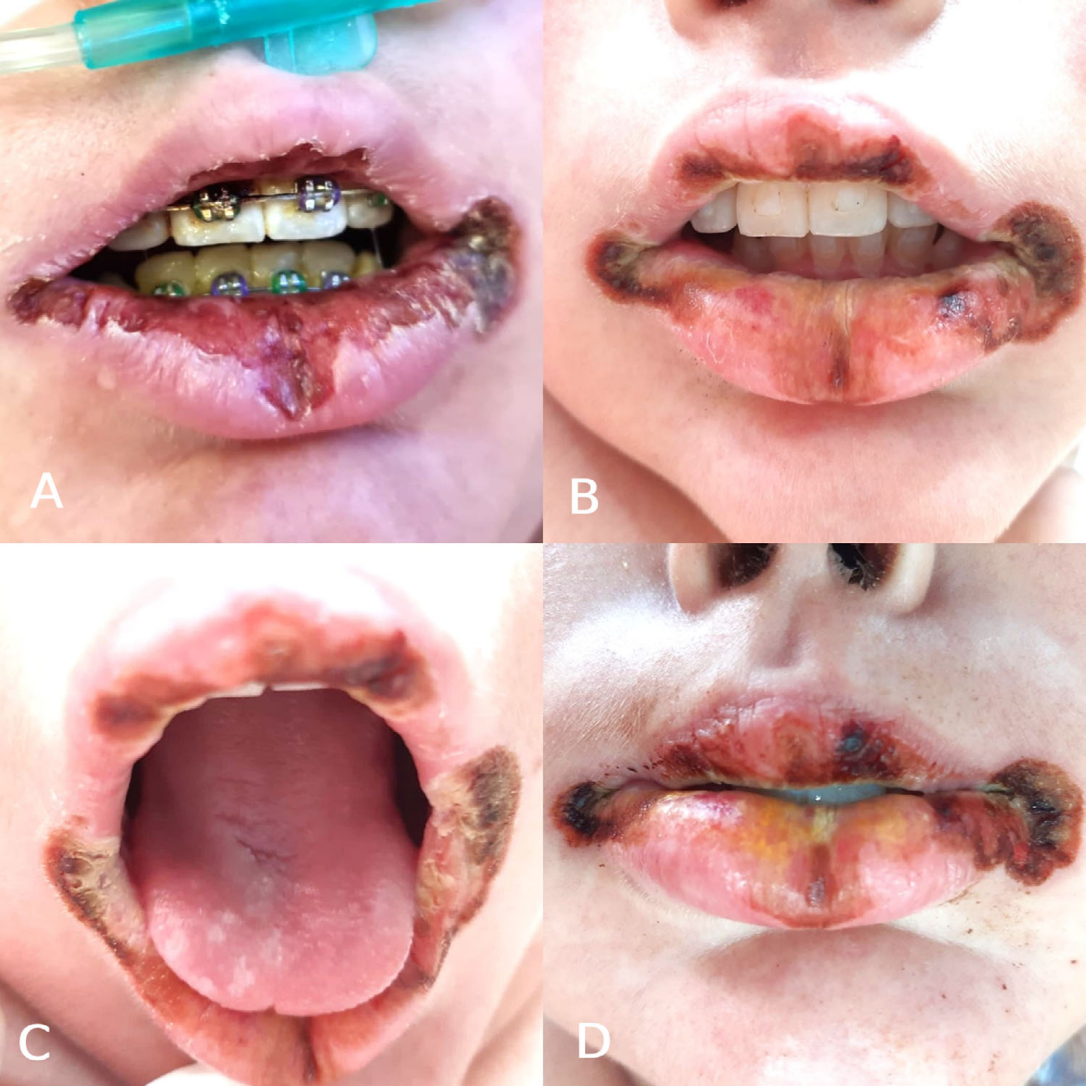
(A) Image from D0: Lip lesions with the presence of biofilm, especially around the orthodontic appliance. (B) Slight improvement in the lower lip lesions, although the wounds on the labial commissure remain almost unchanged. (C)–(D) Images from D + 6: Mild improvement in the lower and upper lip lesions; however, the appearance of the lesions on the labial commissure remains practically unchanged.

On D0, the patient underwent an oral evaluation. Intraoral examination revealed significant biofilm accumulation around the fixed orthodontic appliance and on enamel surfaces (Figure 1A). Diffuse ulcerations with crust formation were observed on both the upper and lower labial mucosa, with more pronounced involvement of the lips. Additionally, nonremovable whitish plaques were noted on the palatal and buccal mucosa, as well as on the gingiva, which appeared erythematous and edematous. The patient reported moderate pain, scoring 5/10 on the pain scale.

At that time, the diagnosis of MIS‐C had not yet been confirmed. Based on the oral findings, differential diagnoses included erythema multiforme, Stevens–Johnson syndrome (SJS), oral manifestations of Kawasaki disease, and viral infections. As part of the initial management, the fixed orthodontic appliance was removed to facilitate hygiene and reduce local trauma (Figure 1B). A PCR swab was collected for cytomegalovirus (CMV), Epstein–Barr virus (EBV), and herpes simplex virus (HSV‐1 and HSV‐2), along with bacterial and fungal cultures. Topical corticosteroid therapy (dexamethasone acetate 1 mg) and antimicrobial photodynamic therapy (aPDT) were initiated. The aPDT protocol included 0.01% methylene blue dye followed by low‐level laser irradiation (100 mW, 660 nm, 6 J, 199.98 J/cm^2^) to assist in local decontamination and symptom relief.

On D + 5, the rheumatology team confirmed the diagnosis of MIS‐C in Children based on the presence of fever, rash, coronary abnormalities (right coronary artery aneurysm < 5 mm), coagulopathy, gastrointestinal symptoms (diarrhea and abdominal pain), elevated ESR/CRP, increased procalcitonin levels, and positive COVID‐19 serology. As part of the therapeutic approach, immunosuppressive therapy with cyclosporine (3 mg/kg/day every 12 h) and methylprednisolone pulse therapy (1 g every 12 h) were prescribed.

During hospitalization, the patient presented with tachypnea, hypertensive episodes, and intermittent tachydyspnea. These clinical manifestations were managed with omeprazole (80 mg/day every 12 h) for gastrointestinal prophylaxis, enalapril (10 mg every 12 h) for blood pressure control, and intravenous albumin for volume expansion and oncotic support. No lymphadenopathy was observed, and renal and hepatic function appeared normal, although confirmation of these findings was pending. Supportive measures also included the transfusion of seven units of platelets in response to severe thrombocytopenia (platelet count: 8000/µL). A chest CT scan revealed subtle ground‐glass opacities in the right upper lobe and lung bases, with no other remarkable findings. Four days before death, the patient developed neurological alterations.

From D + 6 onward, the patient's general condition worsened, with the development of warm, edematous extremities, progressive skin lesions, hypotension, and respiratory distress. Oral manifestations also advanced, with persistent nonremovable whitish plaques on the tongue, worsening crusts on the lips, and white ulcer bases without bleeding (Figure 1C,D). Due to corticosteroid therapy and immunosuppression, the patient developed angular cheilitis.

On D + 7, PCR confirmed HSV‐1 infection. Antiviral therapy with acyclovir was initiated at a dose of 5 mg/kg every 8 h, later adjusted to 500 mg/m^2^ every 8 h. Consequently, topical corticosteroids were discontinued, and aPDT was maintained, alongside lip hydration as part of the decontamination protocol.

**FIGURE 2:**
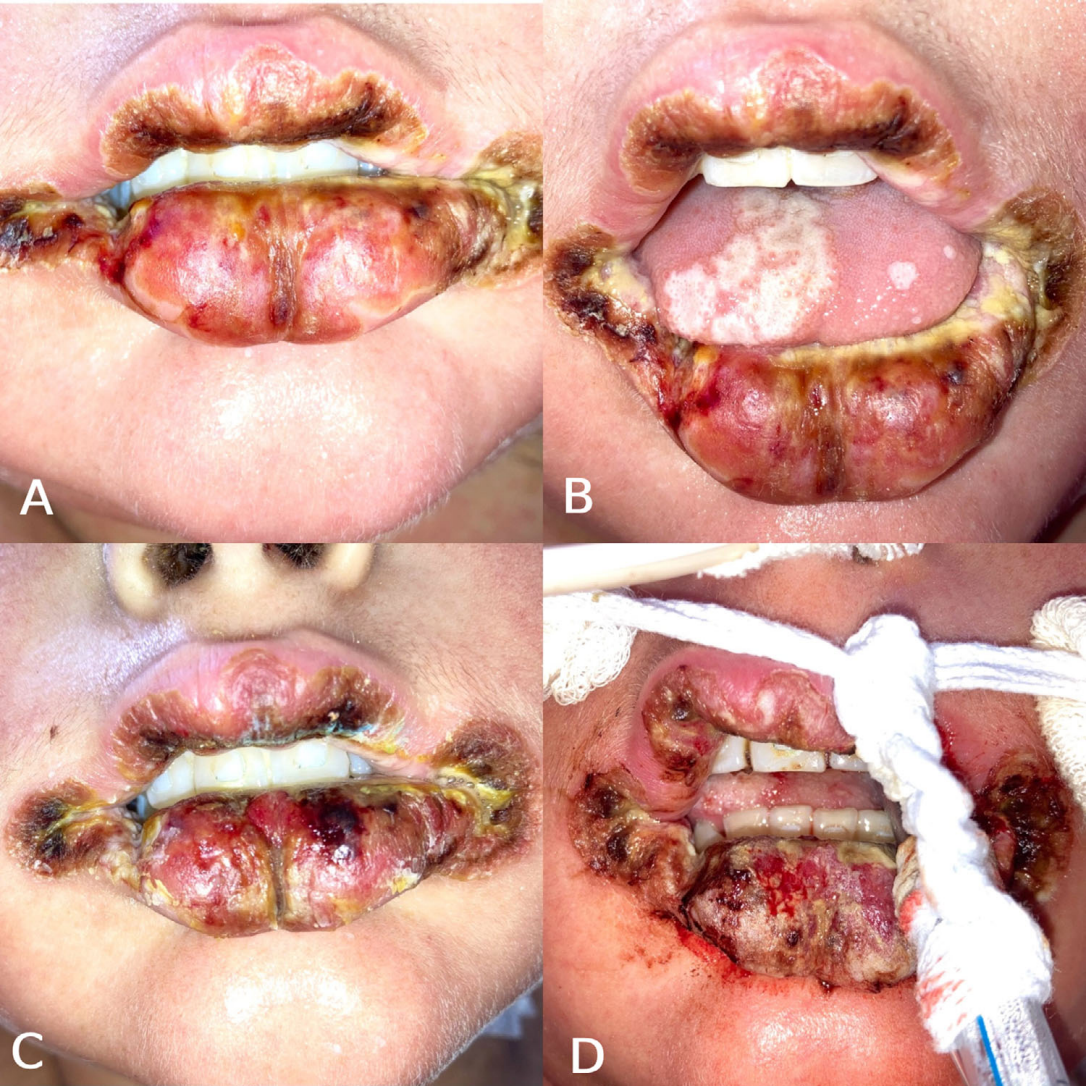
(A)–(B) Images from D + 10 showing worsening of the lesions, especially on the labial commissure. A whitish plaque is also observed on the labial semimucosa and dorsal tongue, which is not removable by scraping. (C) Image from D + 13. (D) Image from D + 16, demonstrating a drastic progression of the lesions on both the lips and the labial commissure.

Despite the withdrawal of corticosteroids, oral lesions progressed, spreading across the tongue and buccal mucosa, with the appearance of extensive white plaques and deep crusting of the lips. On D + 10, the patient reported severe oral pain, making solid food intake difficult. He presented with deep fissures at the lip commissures and the midline of the lower lip, and more defined tongue lesions, characterized by erythematous halos and necrotic white centers (Figure 2A,B). Pain management included intravenous morphine (10 mg) and dipyrone (1 g). Due to signs of fluid overload, furosemide was initiated at a dose of 20 mg every 12 h, and the patient received one unit of packed red blood cells. Hemoculture showed growth of Gram‐positive cocci. As the patient was already under broad‐spectrum antimicrobial coverage—including vancomycin, cefepime, metronidazole, prophylactic fluconazole (30 mg/kg/day), sulfamethoxazole‐trimethoprim (10 mL every 12 h), and albendazole (400 mg/day)—antimicrobial escalation was not immediately necessary. However, due to disease progression, it was recommended to complete five days of methylprednisolone pulse therapy, followed by maintenance with methylprednisolone at 1 mg/kg/dose every 12 h, in addition to initiating intravenous cyclosporine (3 mg/kg/day every 12 h). Supportive care also included amlodipine (5 mg once daily) for blood pressure control and daily intravenous vitamin K administration.

From D + 12 onward, the oral care protocol was maintained with aPDT sessions interspersed with low‐level red light laser therapy (1 J/point) to aid tissue repair and promote analgesia. On that day, the patient experienced cardiovascular deterioration, with reduced ejection fraction (45%), diastolic dysfunction, persistent pericardial effusion, neurological alterations, psychomotor agitation, and melena, along with oral mucosal bleeding (Figure 2C). Orotracheal intubation became necessary due to respiratory decompensation.

Given the clinical deterioration, therapeutic optimization was recommended. The cyclosporine dose was increased to 4 mg/kg (140 mg every 12 h intravenously), and infliximab was introduced at 10 mg/kg (700 mg IV) with premedication including antihistamines, corticosteroids, and antipyretics. Despite remaining hemodynamically stable, the patient continued to present systemic arterial hypertension—managed by increasing the enalapril dose and adding amlodipine—and significant fluid overload with peripheral edema. Effective diuresis was noted, yet a positive and cumulative fluid balance (SVA: 19%) persisted. Furosemide dosing was increased to 60 mg every 6 h, with the addition of spironolactone (25 mg every 12 h) for diuretic synergy.

After a multidisciplinary meeting (involving hematology, rheumatology, nephrology, and the ICU team), vancomycin, metronidazole, cefepime, and sulfamethoxazole‐trimethoprim were discontinued (after completing 7 days or due to potential myelotoxicity). Prophylactic levofloxacin was initiated, and fluconazole was maintained. Daily IV vitamin K supplementation was continued.

On D + 14, the patient remained clinically unstable. The cyclosporine dose was increased to 5 mg/kg/day IV (200 mg every 12 h). Given the partial response to previous therapies, a multidisciplinary team—comprising hematology, rheumatology, nephrology, and the intensive care unit—opted to initiate the HLH‐2004 protocol, which includes dexamethasone, etoposide, and high‐dose cyclosporine.

On D + 17, the patient developed hypotension, likely secondary to dexmedetomidine hydrochloride, requiring norepinephrine and shock‐dose hydrocortisone. The oral lesions became more extensive, with deep bleeding fissures at the lip commissures (Figure 2D).

On D + 18, the patient experienced sudden bradycardia and gasping respiration. Despite all resuscitative efforts, he died.

## 3 Discussion

MIS‐C is a poorly understood condition that is rarely reported in the literature, making it challenging to establish precise criteria for its characterization and diagnosis—key factors for a favorable prognosis [1]. Therefore, distinguishing the signs and symptoms of MIS‐C from those of other conditions is crucial, particularly regarding oral manifestations, which may have multiple etiologies.

In the present case, the patient exhibited persistent fever, diarrhea, conjunctival hyperemia, rash, oral lesions, and a reactive IgG test for SARS‐CoV‐2, along with other cutaneous and gastrointestinal manifestations—findings consistent with previous studies [1, 2, 3]. Notably, cardiovascular involvement was observed, as evidenced by arterial hypertension. This aligns with the findings of Hoste et al. [1], who identified a high prevalence of cardiac complications—including reduced left ventricular ejection fraction—in children with MIS‐C.

Regarding oral manifestations, the patient presented with progressive lip and tongue lesions, with more pronounced involvement of the lip commissures. This pattern—characterized by cracked and crusted lips—was also reported by Nascimento et al. [3] and Halepas et al. [2], who highlighted the scarcity of data on the progression of these lesions due to the limited involvement of dental professionals in hospitalized patient assessments. The lack of consistent data complicates accurate diagnosis and underscores the need for multidisciplinary protocols.

Although syndromes such as Kawasaki disease, SJS, and erythema multiforme can present with similar oral signs, this patient's findings strongly suggest an association with HSV‐1 infection, confirmed by PCR. In Kawasaki disease, which primarily affects children under 5 years old, diagnosis is based on fever (≥ 5 days) and at least four of the following criteria: conjunctival injection, polymorphous rash, extremity changes (edema/erythema), cervical lymphadenopathy, and mucosal changes (erythema/cracking of lips, “strawberry tongue,” mucositis) [4, 5]. In this case, although lip fissures and erythema were observed, classic signs such as the “strawberry tongue” were absent, making Kawasaki disease an unlikely sole explanation for the oral signs.

Stevens–Johnson syndrome is a rare, severe drug‐induced mucocutaneous reaction characterized by epidermal and mucosal detachment. Systemic immunomodulatory therapies, such as cyclosporine and TNF‐α inhibitors like etanercept, have shown favorable outcomes in pediatric cases, including faster re‐epithelialization and lower mortality rates [6, 7]. In the present case, however, increasing cyclosporine to 5 mg/kg/day (200 mg every 12 h IV) failed to improve oral lesions, supporting that the clinical presentation was not consistent with Stevens–Johnson syndrome. The absence of skin involvement and blistering mucosal lesions further ruled out this diagnosis.

Erythema multiforme is an inflammatory immune reaction often triggered by viral infections, particularly HSV‐1 and 2, manifesting with swollen, cracked, and crusted lips, as well as lesions on nonkeratinized mucosa [8]. However, the predominant signs in this patient—cracked, crusted lip lesions with minimal tongue involvement—are more characteristic of HSV‐1‐associated complications.

The confirmation of HSV‐1 in the mucosa and the observed pattern of oral lesions suggest that, in the context of immunosuppression induced by both MIS‐C and its treatment, HSV‐1 reactivation occurred more aggressively, exacerbating the lesions. A healthy oral cavity depends on the integrity of the epithelial barrier, adequate salivary flow, salivary components, and the protective effects of the oral microbiota. When these defense mechanisms are compromised, opportunistic infections like HSV‐1 can progress aggressively. Current guidelines highlight that in immunocompromised patients, HSV‐1 reactivation may present severely, often requiring early systemic antiviral therapy. Accordingly, on D + 7, intravenous acyclovir was initiated at 5 mg/kg every 8 h, later adjusted to 500 mg/m^2^, aligning with recommendations for patients with extensive mucosal involvement or impaired oral intake. This therapeutic choice was appropriate given the severity of the lesions and systemic vulnerability, prioritizing rapid viral control to prevent further tissue damage and complications. While topical or oral antivirals are effective in mild cases, intravenous administration remains the standard in more severe or refractory presentations such as this [9]. Additionally, HSV‑related infections are highly prevalent worldwide, with an estimated 64% of individuals under 50 years of age infected with HSV‑1. This virus typically establishes latent infection in the oral mucosa and peripheral nerves, remaining dormant for most of the host's life. In immunocompromised patients, reactivation is often severe and may involve both keratinized and nonkeratinized oral mucosa, leading to clinically significant lesions and complications [10].

At hospital admission, the patient presented with lip and lip commissure lesions, along with a whitish lesion on the dorsal tongue, a pattern that worsened as his clinical condition progressed. These findings reinforce that, while the patient was diagnosed with MIS‐C, which can cause oral manifestations, the use of multiple systemic medications may have contributed to lesion exacerbation, both by intensifying the inflammatory process and by promoting immunosuppression, thereby allowing viral and bacterial proliferation in the oral cavity.

The combination of clinical findings highlights that appropriate management of MIS‐C requires not only accurate diagnosis of the systemic condition but also the identification and targeted treatment of oral complications. Prior knowledge of the specific characteristics of MIS‐C, combined with expertise in managing oral infections in immunocompromised patients, is essential to prevent lesion progression and ensure more effective treatment.

## 4 Conclusion

Despite the current control of COVID‐19 due to widespread vaccination, the virus remains a major trigger for MIS‐C—a condition that, as demonstrated in this case, can have fatal consequences. MIS‐C frequently presents with early oral manifestations; however, these changes can also be observed in other syndromes, such as Kawasaki disease, or result from systemic changes in critically ill patients. Thus, the correct identification of oral manifestations in MIS‐C is essential for healthcare professionals to implement the most appropriate treatment, ultimately contributing to improved patient prognosis.

## Conflicts of Interest

The authors declare no conflicts of interest.
